# Supplementary material for: Age at natural menopause and its determinants in female population of Kharameh cohort study: Comparison of regression, conditional tree and forests
Source: PLoS One. 2024 Apr 16;19(4):e0300448. doi: 10.1371/journal.pone.0300448 (PMC11020934; doi:10.1371/journal.pone.0300448)
Supplement: S1 Checklist — (DOCX) [file pone.0300448.s001.docx]

**Human Participants Research Checklist**

***Complete the following if your study involved human participants or human participants’ data. These questions should be addressed for prospective and retrospective studies.***

1. Did you obtain ethics approval for this study?
   - If yes, please upload (file type “Other”) the original approval document you received from your ethics committee. If the original document is in another language, please also provide an English translation.

___ Uploaded	___ N/A

- If you did not obtain ethical approval, please explain why this was not required below.

Author response: Yes, this study obtained ethics approval (IR.SUMS.SCHEANUT.REC.1400.104). we uploaded the approval document we received from the ethics committee.

2. If you prospectively recruited human participants for the study – for example, you conducted a clinical trial, distributed questionnaires, or obtained tissues, data or samples for the purposes of this study, please report in the Methods:
   i. the day, month and year of the **start and end** of the recruitment period for this study.
   ii. whether participants provided informed consent, and if so, what type was obtained (for instance, written or verbal, and if verbal, how it was documented and witnessed). If your study included minors, state whether you obtained consent from parents or guardians. If the need for consent was waived by the ethics committee, please include this information.

___ Completed	___ N/A

Author response: The data is not collected for the current study specifically. Actually, we have used a part of the data collected in phase one of the PERSIAN Kharameh cohort study. The PERSIAN Cohort Study started in Kharameh in October 2014, and enrollment ended in February 2017. In the Kharameh cohort study, written informed consent was obtained from each participant. The ethics approval code of the Kharameh cohort study is “IR.SUMS.REC.1393.S7421”. Further information has been mentioned in “Prospective Epidemiological Research Studies in Iran (the PERSIAN Cohort Study): Rationale, Objectives, and Design” (Accession Number: 29145581 PMCID: PMC6279089 DOI: 10.1093/aje/kwx314)

3. If you are reporting a retrospective study of medical records or archived samples, please report in the Methods section:
i. the day, month and year when the data were accessed for research purposes
ii. whether authors had access to information that could identify individual participants during or after data collection

___ Completed	___ N/A

Author response: The data reached us anonymously after approval by the research committee in February 2022. Only one code was assigned to each participant and the research team was not able to identify the participants through that code in the data analysis of this study.
