# Supplementary material for: Age at natural menopause and its determinants in female population of Kharameh cohort study: Comparison of regression, conditional tree and forests
Source: PLoS One. 2024 Apr 16;19(4):e0300448. doi: 10.1371/journal.pone.0300448 (PMC11020934; doi:10.1371/journal.pone.0300448)
Supplement: Attachment — Submitted filename: comments.docx [file pone.0300448.s002.docx]

This is an interesting, well-written article with excellent methodology that examines factors affecting ANM using various machine learning methods. In my opinion, this article is suitable for publication in PLoS ONE, just correct the following points in the modified version.

1. What is meant by natural menopause? Please explain it in the abstract section.
2. The authors wrote in the introduction that ethnicity, region, socioeconomic position, environment, life style, occupation, education, residence, and marital status have an effect on menopause. Please specify which subgroups of these variables have a positive effect on premature menopause.
3. The phrase " As can be seen, breastfeeding has little impact on ANM and this statistical significance may be due to the large sample size." should be transferred to the discussion in the findings section.
